# Amphibian (*Xenopus laevis*) Tadpoles and Adult Frogs Differ in Their Antiviral Responses to Intestinal Frog Virus 3 Infections

**DOI:** 10.3389/fimmu.2021.737403

**Published:** 2021-08-20

**Authors:** Kelsey A. Hauser, Julia C. Singer, Muhammad Riadul H. Hossainey, Tyler E. Moore, Emily S. Wendel, Amulya Yaparla, Namarta Kalia, Leon Grayfer

**Affiliations:** Department of Biological Sciences, George Washington University, Washington, DC, United States

**Keywords:** amphibian, ranavirus, intestine, myeloid cells, interferons

## Abstract

The global amphibian declines are compounded by ranavirus infections such as Frog Virus 3 (FV3), and amphibian tadpoles more frequently succumb to these pathogens than adult animals. Amphibian gastrointestinal tracts represent a major route of ranavirus entry, and viral pathogenesis often leads to hemorrhaging and necrosis within this tissue. Alas, the differences between tadpole and adult amphibian immune responses to intestinal ranavirus infections remain poorly defined. As interferon (IFN) cytokine responses represent a cornerstone of vertebrate antiviral immunity, it is pertinent that the tadpoles and adults of the anuran *Xenopus laevis* frog mount disparate IFN responses to FV3 infections. Presently, we compared the tadpole and adult *X. laevis* responses to intestinal FV3 infections. Our results indicate that FV3-challenged tadpoles mount more robust intestinal type I and III IFN responses than adult frogs. These tadpole antiviral responses appear to be mediated by myeloid cells, which are recruited into tadpole intestines in response to FV3 infections. Conversely, myeloid cells bearing similar cytology already reside within the intestines of healthy (uninfected) adult frogs, possibly accounting for some of the anti-FV3 resistance of these animals. Further insight into the differences between tadpole and adult frog responses to ranaviral infections is critical to understanding the facets of susceptibility and resistance to these pathogens.

## Introduction

Amphibian populations around the world face severe population declines and extinction, threatening not only species diversity but also ecosystem-level stability. Infections by ranavirus genus members (family *Iridoviridae*) such as Frog Virus 3 (FV3), are significant contributors to these events (1–3). The tadpoles of anuran amphibians are considerably more adversely affected by ranavirus infections than adult frogs (4–8). However, the mechanisms dictating amphibian tadpole and adult immune susceptibility or resistance to ranaviruses remain poorly understood, precluding the development of effective means to counteract these catastrophic events.

The *Xenopus laevis* frog represents the most extensively used model of amphibian host-ranavirus interactions, with past studies indicating that compared to adult frogs, the tadpoles of this species are less effective at mounting proinflammatory responses to FV3 (9). Nonetheless, tadpoles do undergo antiviral cytokine responses to this pathogen, albeit with distinct responses than adult frogs (10–12). Moreover, despite their greater likelihood of succumbing to FV3, infected *X. laevis* tadpoles tend to bear lower viral loads than adult frogs across several key virally targeted tissues (5, 11).

Across vertebrates, antiviral interferon (IFN) cytokines serve as the cornerstone of antiviral immune responses (13). More recently diverged species including reptiles, birds and mammals encode three types of IFNs (14), wherein type I and type III IFNs are thought to be more prominently involved in antiviral responses (13). These type I and type III IFNs are encoded by intronless and intron-containing (five exon/four intron pre-mRNAs) transcripts, respectively (13). Conversely, bony fish lack type III IFNs and encode intron-containing type I IFNs with five exon/four intron organization (13). Because amphibians encode intronless and intron-containing type I and type III IFNs, they represent a key stage in the evolution of vertebrate IFN cytokine families (15, 16). Although the functional roles of these amphibian cytokines remain to be fully explored, as alluded to above, *X. laevis* tadpoles and adult frogs respond to FV3 by upregulating distinct type I and type III IFN genes (10, 11, 17). Moreover, we showed *X. laevis* possess granulocyte and macrophage populations that are key producers of these antiviral agents (18, 19).

Presently, we compare the *X. laevis* tadpole and adult frog antiviral responses to FV3 in their intestines, which represent a major site of viral entry.

## Materials and Methods

### Animals

Outbred mixed-sex tadpole (Nieuwkoop and Faber; NF Stage ~54) and adult (~1 year-old) *X. laevis* were purchased from the Xenopus 1 Facility (Dexter, Michigan, USA). Animals were housed and handled under strict laboratory and IACUC regulations (Approval number 15-024).

### Cell Culture Media and Conditions

All cell cultures were established using Iscove’s Modified Dulbecco’s Medium (Sigma-Aldrich, St. Louis, Missouri, USA) supplemented with 10% fetal bovine serum, 0.25% *X. laevis* serum, insulin (Sigma), non-essential amino acids (Sigma), and primatone (2.5%). This medium contained 10 μg/ml Gentamycin (Thermo Fisher Scientific, Waltham, Massachusetts, USA) and 100 U/ml penicillin/100 μg/ml streptomycin (Gibco, Thermo Fisher Scientific), was buffered with sodium bicarbonate to pH 7.7, and diluted to 1 in 5 parts with water to amphibian osmolarity. All leukocyte cultures were grown at 27°C with 0.5% CO_2_. Amphibian phosphate buffered saline (A-PBS) consisted of 100 mM sodium chloride, 8 mM sodium phosphate, 1.5 mM potassium phosphate; pH 7.7.

### Intestine Tissue and Cell Isolation

After euthanasia, whole tadpole and adult intestines were excised from animals using sterilized dissection tools. Tissues taken for histology were processed as described below. For cell isolation, whole intestines were incubated in Liberase (0.1mg/ml, Roche Diagnostics) diluted with A-PBS for 30 minutes at 27°C and then washed 1x with A-PBS. Cells used for staining were further passed through a 70 μm cell strainer (VWR, Radnor, Pennsylvania, USA) to produce single-cell suspensions. The viability of the isolated intestinal cell was confirmed by Trypan blue exclusion.

### Histology and Cell Staining

*X. laevis* intestines excised for histology were immediately fixed in 10% neutral buffered formalin (VWR) for 24 hours. Intestines were processed and embedded in paraffin, sectioned (5 um) by the GWU Pathology Core. Sections were then stained with Naphthol AS-D Chloroacetate (Specific Esterase; Sigma) or α-Naphthyl Acetate (Non-Specific Esterase; Sigma) according to the manufacturer’s instructions and optimized to frog tissues.

Alternatively, intestinal single cell suspensions were cytocentrifuged onto glass microscope slides (VWR), fixed in 10% neutral buffered formalin for 30 minutes, and stained as above. Where appropriate, tissues and cells were also counterstained with hematoxylin (Sigma) (tissues: diluted 1 in 3 parts with A-PBS; cells: diluted 1 in 5 parts with A-PBS).

### FV3 Stocks, Animal, and Cell Infections

FV3 *(FV3; wild type, ATCC VR-567)* production has been described previously (20). Briefly, baby hamster kidney (BHK-21) cells were inoculated with FV3 (multiplicity of infection; MOI: 0.1), grown at 5% CO_2_ and 30°C for 5 days or until the cells were completely lysed. The supernatants containing FV3 were cleared by ultracentrifugation, collected over 30% sucrose, and resuspended in A-PBS. Viral titers were determined using plaque assay analysis over BHK-21 cells.

Tadpoles (*N*=5-6) and adult frogs (*N*=5-6) were infected by water bath with 10^6^ plaque forming units (PFU) of FV3 in 100 ml of water for 1 hour before being transferred into FV3-free water and infections permitted to proceed for an additional 5 hours. Cohort animals were mock infected with FV3-free water in otherwise identical conditions. Animals were euthanized by tricaine mesylate overdose (tadpoles: 1%; adult frogs: 5%), intestines excised for further processing.

For all *in vitro* infection studies, 10^4^ control intestinal cells (*N*=5-6 tadpoles/adults) were infected with a multiplicity of infection (MOI) of 0.5 for 6 hours, incubated in the medium described above at 27°C with 5% CO_2_. Subsequently, the cells were trypsinized to remove attached but not internalized virus and washed with A-PBS before being flash-frozen in Trizol reagent (Invitrogen) over dry ice and stored at -20°C until RNA and DNA isolation.

### Production of the *X. laevis* Recombinant CSF-1 and IL-34

The production of *X. laevis* recombinant (r) rCSF-1 and rIL-34 has been previously described (19). Briefly, the *X. laevis* CSF-1 and IL-34 sequences representing the signal peptide-cleaved transcripts were ligated into the pMIB/V5 His A insect expression vectors (Invitrogen) and transfected into Sf9 insect cells (cellfectin II, Invitrogen). Recombinant protein production was confirmed by western blot and the positive transfectants were selected using 10 μg/mL blasticidin. The expression cultures were scaled up as 500 ml liquid cultures, grown for 5 days, pelleted, and the supernatants collected. These were dialyzed overnight at 4°C against 150 mM sodium phosphate, concentrated against polyethylene glycol flakes (8 kDa) at 4°C, dialyzed overnight at 4°C against 150 mM sodium phosphate, and passed through Ni-NTA agarose columns (Qiagen). Columns were washed with 2 × 10 volumes of high stringency wash buffer (0.5% Tween 20; 50 mM Sodium Phosphate; 500 mM Sodium Chloride; 100 mM Imidazole) and 5 x 10 volumes of low stringency wash buffer (as above, but with 40 mM Imidazole). Recombinant cytokines were eluted using 250 mM imidazole and were confirmed by western blot against the V5 epitopes on the proteins and the protein concentrations were determined by Bradford protein assays (BioRad). Halt protease inhibitor cocktail (containing AEBSF, aprotinin, bestatin, E-64, leupeptin and pepstatin A; Thermo Scientific) was added to the purified proteins, which were then stored at -20°C in aliquots until use.

### Chemotaxis Assays

Blind well Boyden chambers (Neuro Probe, Gaithersburg, Maryland, USA) were used for chemotaxis assays. The bottom wells were loaded with rCSF-1 or rIL-34 (10 - 10^-6^ ng/ml) in 100 μl of media, overlaid with 13 mm chemotaxis filters (5 μm pore size; Neuro Probe), and the top wells were loaded with isolated intestinal cells from FV3-infected tadpoles in 100 μl of media. Chemokinesis assays were performed by loading the most chemoattractive concentration to both the bottom and top wells, thus abolishing any chemoattractant gradients. The Boyden chambers were incubated for 3 hours at 27°C and 5% CO2 at which time the top wells were aspirated and the filters wiped with cotton swabs. The bottom sides of the filters were stained with Giemsa (Gibco, Thermo Fisher). To quantify migrating cells, 10 random fields of view were counted for each filter under a 40x objective. Chemoattracted cells were also collected from the bottom wells and flash frozen in Trizol reagent for further gene expression analyses.

### Isolation of RNA and DNA From Cells and Tissues

For all experiments, tadpole and adult cells or intestine tissues from FV3-infected animals were homogenized by passage through progressively higher gauge needles in Trizol reagent (Invitrogen, Carlsbad, California, USA), flash frozen on dry ice and stored at -80°C until RNA and DNA isolation. RNA isolation was performed using Trizol (Invitrogen) according to the manufacturer’s directions. DNA was isolated from the Trizol following RNA isolation. In brief, following phase separation and extraction of RNA, the remaining Trizol layer was mixed with back extraction buffer (4 M guanidine thiocyanate, 50 mM sodium citrate, 1 M Tris pH 8.0), and centrifuged to isolate the DNA containing aqueous phase. The DNA was precipitated overnight with isopropanol, pelleted by centrifugation, and washed with 70% ethanol and resuspended in TE (10 mM Tris pH 8.0, 1 mM EDTA) buffer. DNA was then purified by phenol: chloroform extraction and resuspended in molecular grade water.

### Tadpole Triiodothyronine Treatments and Pharmacological Inhibition Studies

Tadpoles were reared in water containing T3 (10 nM final concentration) or in water containing diluted solvent control (NaOH) alone for 5 days (21). During this time, subsets of animals also received daily intraperitoneal administration of a CSF-1R inhibitor (GW-2580; 100 mg/kg body weight (22); Apex Bio), a CXCR1/2 inhibitor (reparixin; 50 mg/kg body weight; Sigma) or vehicle control (18). The same injection sites were used for the daily drug administrations, and the water was changed daily for the 5-day duration of the experiments.

### Quantitative Analysis of Gene Expression and FV3 Copy Number

Quantitative analysis of *X. laevis* gene expression and FV3 viral copy number has been described (23–25). All cDNA syntheses were performed using iScript cDNA synthesis kits in accordance with manufacturer’s directions (Bio-Rad, Hercules, CA) and using 500 ng of total RNA. Quantitative qRT-PCR analysis was performed using 2.5 µl of the derived cDNA templates.

FV3 viral loads were determined by absolute qPCR and performed using 50 ng of total isolated DNA and compared against a serially diluted standard curve. In brief, the FV3 standard curve was derived by serially diluting a pGEM-T plasmid bearing an FV3 vDNA Pol (ORF 60R) fragment into 10^1^-10^8^ vDNA Pol fragment-containing copies. These were used as the standard curve templates absolute qPCR assays.

All experiments were performed using the CFX96 Real-Time System (Bio-Rad Laboratories, Hercules, California, USA) and iTaq Universal SYBR Green Supermix (Bio-Rad Laboratories). The BioRad CFX Manager software (SDS) was employed for all expression analysis. All primers were validated prior to use (**Supplementary Table 1**).

### Statistical Analysis

Statistical analyses was performed using a one-way or two-way analysis of variance (ANOVA) and *post hoc* t-test, using Vassar Stat (http://faculty.vassar.edu/lowry//anova1u.html) and Graph Pad (https://www.graphpad.com/quickcalcs/ttest1.cfm) statistical programs, respectively. Probability level of *p*< 0.05 was considered significant.

## Results

### FV3-Infected Tadpoles Possess Lower Intestinal Viral Loads Than Adult Frogs

**Figure 1:**
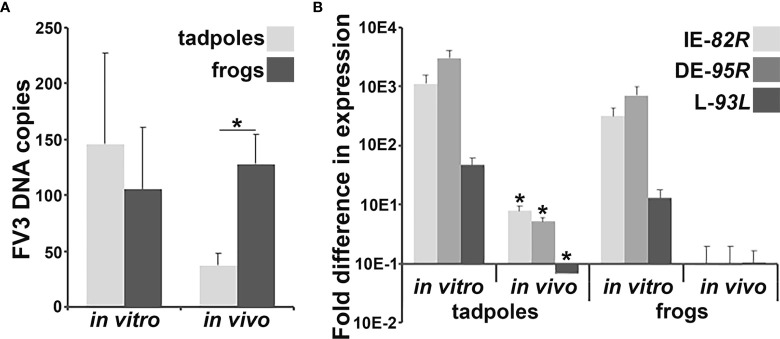
Analyses of *in vivo* and *in vitro* FV3 DNA loads and gene expression in tadpole and adult frog intestines. Tadpoles (*N* =6) and adult frogs (*N* = 5) were infected by water bath with 10^6^ PFU of FV3 for 6 hrs and their intestinal **(A)** FV3 loads and **(B)** FV3 gene expression examined. Alternatively, tadpole and adult frog intestinal cells (*N* = 6) were infected *in vitro* with FV3 (0.5 MOI) and the **(A)** FV3 DNA loads and **(B)** expression of 82R (immediate early; IE), 95R (delayed early; DE) and 93L (late; L) FV3 genes, assessed by qPCR. The results are means ± SE of viral loads or viral gene expression. Asterisks above lines (*; **A**) denote statistical differences between the treatment groups denoted by the line and asterisks (*; **B**) denote statistically significant difference from *in vitro* expression, *p* < 0.05.

We previously observed that following water bath challenge with FV3, *X. laevis* tadpoles possessed significantly lower viral loads in their skin tissues than adult frogs (11). Since the gastrointestinal tracts of these animals represent another potential site of FV3 entry, we examined the intestines of tadpoles and adult frogs 6 hours after water bath challenge with FV3 for viral DNA loads and gene expression (infection time was chosen based on preliminary *ifn* gene expression studies). Our past studies indicated that FV3 infections result in rapid immune cell recruitment, thereby affecting the viral infection outcomes (12). Accordingly, we compared the FV3 DNA loads and viral gene expression in virally challenged animals to *in vitro*-infected tadpole and adult frog intestinal cells, which would not be subject to the effects of incoming immune populations. Consistent with our previous findings with FV3-infected tadpole and adult skins, FV3-challenged tadpoles possessed significantly lower viral loads in their intestines than adult frogs (**Figure 1A**). Conversely, there were no differences in FV3 loads between the tadpole and adult frog intestinal cells infected with FV3 *in vitro* (**Figure 1A**). The FV3 loads were greater in *in vitro*-challenged tadpole intestinal cells than those seen in *in vivo* FV3-challenged tadpole intestines (**Figure 1A**). We did not see differences between the *in vitro-* and *in vivo*-challenged adult frog intestinal FV3 loads (**Figure 1A**).

To determine the extent of viral replication within the *X. laevis* tadpole and adult frog intestinal cells infected *in vitro* or *in vivo*, we examined the respective cells and tissues for their expression of FV3 82R immediate early (IE), 95R delayed early (DE) and 93L late (L) genes (**Figure 1B**). *In vitro* FV3-challenged tadpole and adult frog intestinal cells had comparable FV3 gene expression, while FV3 gene expression was substantially lower in *in vivo*-challenged animals, significantly so for tadpoles but not adults (**Figure 1B**). While *in vivo* FV3-challenged adult frog intestinal cells possessed relatively low viral gene expression compared to the *in vitro*-challenged frog cells, the *in vivo* FV3 gene expression was highly variable and thus not statistically different from the *in vitro*-infected frog cells (**Figure 1B**).

### Tadpoles Mount More Pronounced Intestinal IFN Gene Responses to FV3

**Figure 2:**
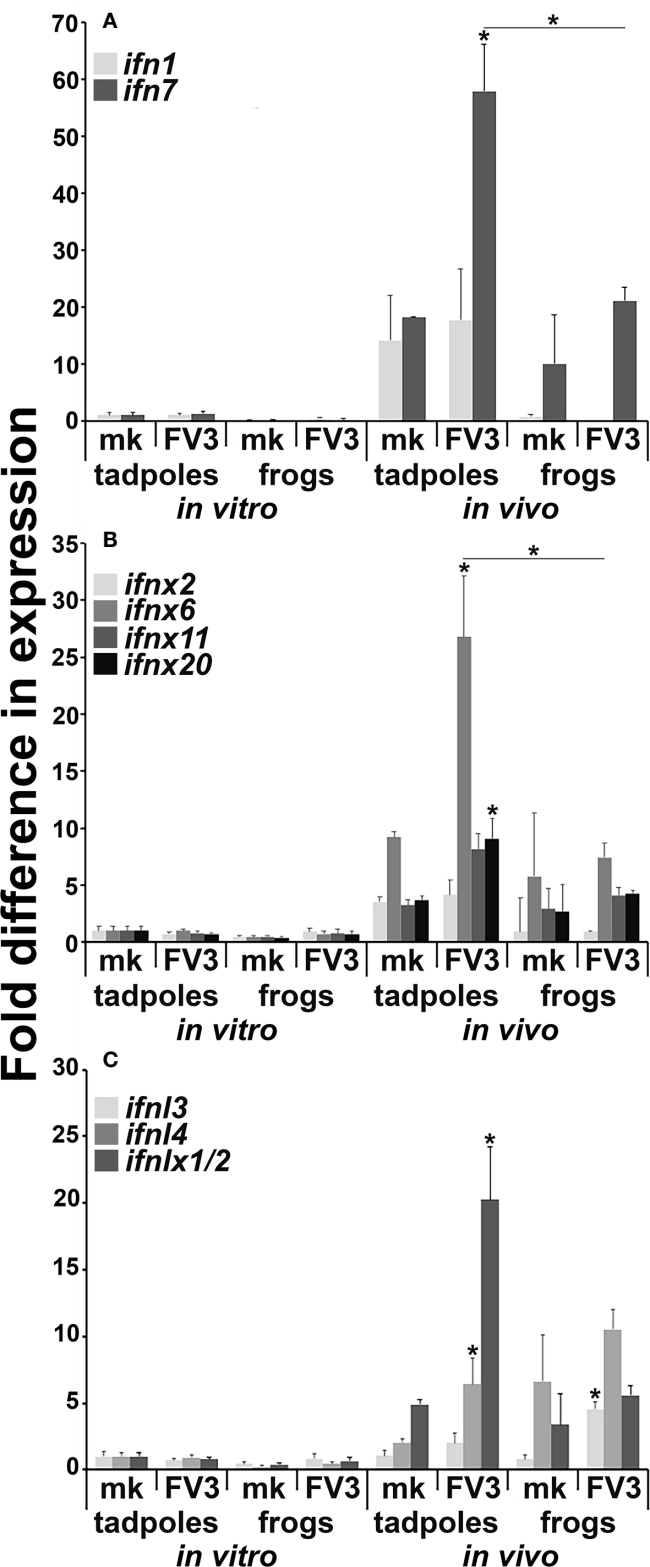
Tadpoles and adult frogs differ in their intestinal antiviral *ifn* gene expression responses to FV3 infections. Tadpoles (*N* =6) and adult frogs (*N*=5) were infected by water bath with 10^6^ PFU of FV3 for 6 hrs. Alternatively, tadpole and adult intestinal cells (*N*=6) were infected *in vitro* with FV3 (0.5 MOI). The expression of **(A)** intron-containing (*ifn*) and **(B)** intronless (*ifnx*) type I IFN genes and **(C)** type III intron-containing (*ifnl*) and intronless (*ifnlx*) IFN genes was examined relative to *gapdh* endogenous control. The results are means ± SE of gene expression. Asterisks (*) denote statistical differences between respective *in vitro* and *in vivo* expression and asterisks above lines (*) denote statistical differences between the treatment groups denoted by the line, *p*<0.05.

We previously reported that *X. laevis* tadpoles and adult frogs mount distinct type I and type III IFN responses to FV3 across several different tissues (10–12). Presently, we examined the gene expression of disparate [based on phylogeny, sequence identity (11)] intron-containing and intronless type I and type III IFN genes (*ifn*, *ifnx*, *ifnl*, *ifnlx*, respectively) in FV3-infected tadpole and adult frog intestines and intestinal cells infected *in vitro* with FV3 (**Figure 2**). Neither tadpole nor adult frog intestinal cells infected *in vitro* with FV3 possessed significantly increased expression of any of the examined *ifn* genes (**Figures 2A–C**). Conversely, *in vivo* FV3-infected tadpoles significantly increased their intestinal expression of *ifn7*, *ifnx6*, *ifnx20*, *ifnl4* and *ifnlx1/2*, while the *in vivo* FV3-challenged adult frogs only exhibited increased intestinal *ifnl3* expression (**Figures 2A–C**). We did not see significant difference in baseline (mock-infected) expression of any of the examined *ifn* genes between tadpole and adult frog intestinal cells (**Figure 2**).

### Tadpoles Recruit Myeloid Cells Into Their FV3-Infected Intestinal Tissues

**Figure 3:**
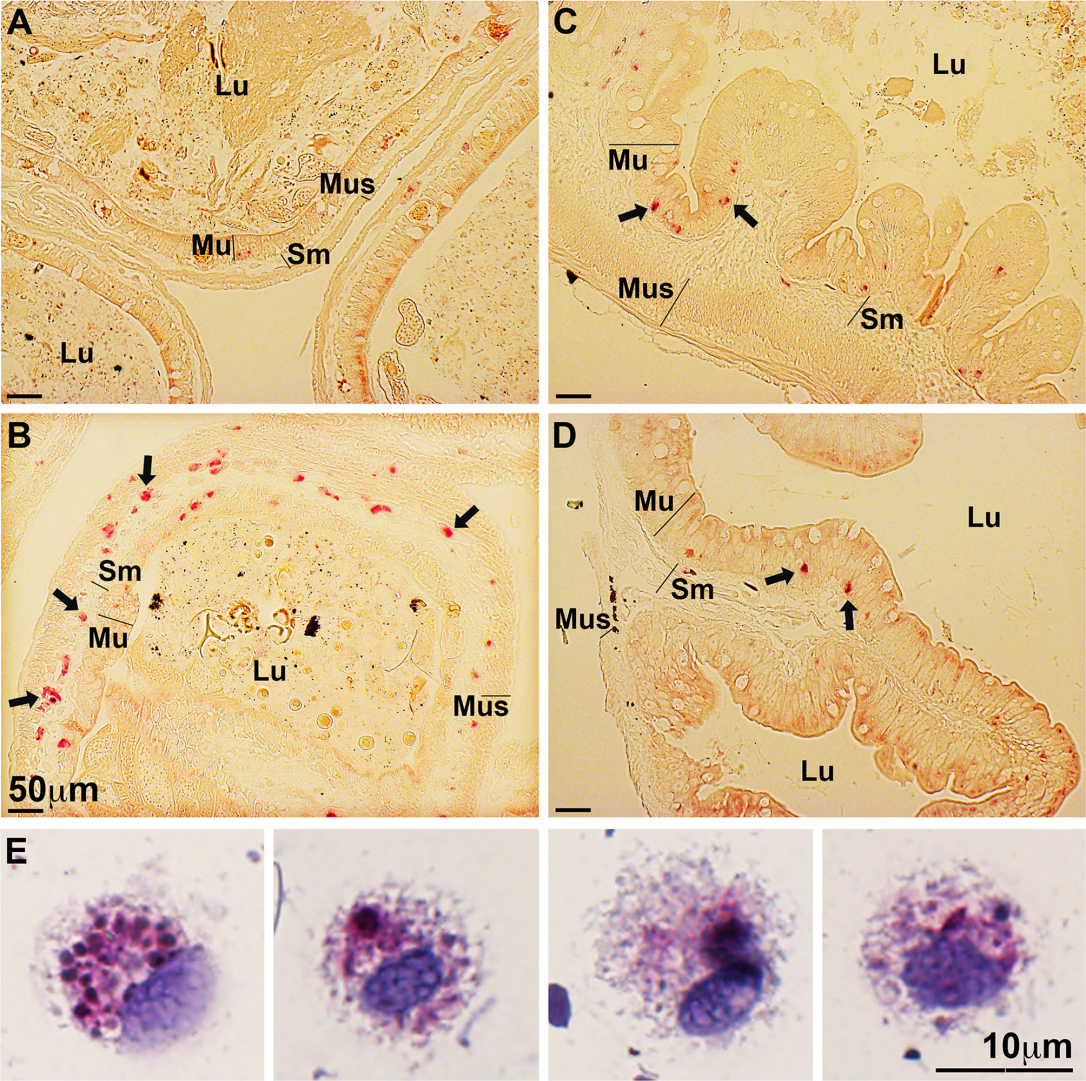
FV3-infected tadpoles recruit esterase-positive myeloid cells into their intestines while adult frog intestines contain resident esterase-positive myeloid cells. Tadpoles and adult frogs were infected by water bath with 10^6^ PFU of FV3 for 6 hrs. Their intestines were examined for the presence of specific esterase-positive cells (*N*=6 per treatment group) using the NASDCl- specific esterase (Leder) stain. **(A)** Mock-infected and **(B)** FV3-infected tadpole intestines. **(C)** Mock-infected and **(D)** FV3-infected adult frog intestines. Lu, lumen; Mu, mucosal; Sm, sub-mucosal r; Mus, muscularis layers. **(E)** Morphology of esterase-positive cells from FV3-infected tadpole intestines. These results are representative of 3 separate experiments.

We previously showed that certain *X. laevis* granulocyte subset(s) and macrophages differentiated by the interleukin-34 (IL-34) but not colony-stimulating factor-1 (CSF-1) macrophage growth factors, are important to these animals’ antiviral defenses and exhibit broad *ifn* gene expression (18, 26–28). Notably, these granulocyte subset(s) and IL-34-macrophages, but not CSF-1-macrophages, possess robust and specific esterase activity (18, 19). Because *in vivo* FV3-challenged tadpoles exhibited increased *ifn* gene expression in their intestines but failed to mount *ifn* responses *in vitro*, we hypothesized that the observed *in vivo ifn* expression responses may be due to infiltrating *ifn*-expressing immune cells such as granulocytes and/or IL-34-derived macrophages. Accordingly, we examined mock- and FV3-infected tadpole and adult frog intestinal tissues for possible presence/recruitment of specific esterase-positive cells (**Figure 3**). While mock-infected tadpole intestines possessed very few specific esterase-positive cells (**Figure 3A**), FV3-infected tadpole intestines showed robust infiltration of specific esterase-positive cells into the mucosa, submucosa, and muscularis layers of their intestinal tissues (**Figure 3B**). By contrast, healthy (mock-infected) adult frogs possessed substantial numbers of specific esterase-positive cells in their intestines, primarily in the mucosa layers (**Figure 3C**). The proportions of these cells were not significantly altered following FV3 infections (**Figure 3D**).

Intuitively, mock-infected tadpoles possess non-specific esterase [NSE; marker of myeloid-lineage cells (29)]-positive cells in their intestines (predominantly in the mucosa layers), and the proportion of these NSE-staining cells increased following FV3-challenge (**Supplementary Figure 1**).

To examine the infiltrating tadpole specific esterase-positive cells further, we repeated the tadpole FV3 infection study and examined specific esterase activity in cell suspensions prepared from the intestines of these animals (**Figure 3E**). We did not observe specific esterase-positive cells with polymorphonuclear granulocyte morphology, suggesting that the specific esterase-positive leukocytes recruited into the infected tadpole intestines are unlikely to be conventional granulocytes. Conversely, these FV3-infected tadpole intestine-derived cell suspensions possessed specific esterase-positive cells bearing monocyte-like mononuclear phagocyte morphologies, reminiscent of the *X. laevis* IL-34-macrophages [(19); **Figure 3E**].

### Myeloid Cell Recruitment in FV3-Infected Tadpoles Corresponds With Increased Expression of Myeloid Markers and Growth Factors

**Figure 4:**
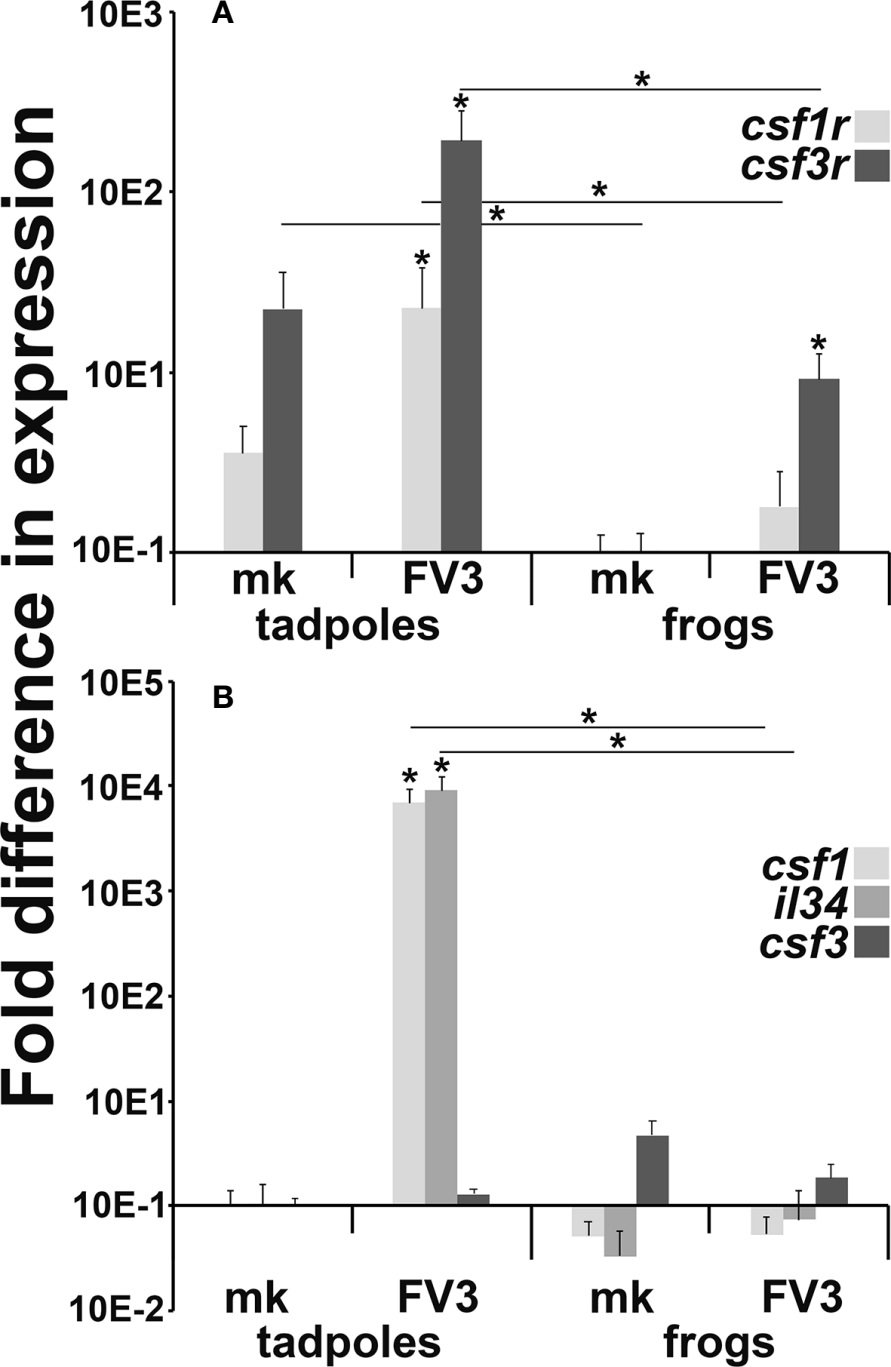
Myeloid cell **(A)** growth factor receptor and **(B)** growth factor gene expression in FV3-challenged tadpole and adult frog intestines. Tadpoles (*N*=6) and adult frogs (*N*=6) were infected by water bath with 10^6^ PFU of FV3 for 6 hrs and their intestinal expression of **(A)** *csf1r*, *csf3r* and **(B)** *csf1*, *il34* and *csf3* was examined relative to *gapdh* endogenous control. The results are means ± SE of gene expression. Asterisks (*) denote statistical differences between mock- and FV3-infected groups and asterisks above lines (*) denote statistical differences between the treatment groups denoted by the line, *p*<0.05.

While the CSF1 receptor (*csf1r*) is a reliable marker of macrophage-lineage cells, the *csf3r* is expressed by both frog granulocytes and IL-34-macrophages (19). To discern if the increased presence of cells bearing frog IL-34-macrophage-like morphology/enzymology in the FV3-infected tadpole intestines was indeed due to increased presence of myeloid cells at this site, we examined the expression of *csf1r* and *csf3r* in mock- and FV3-infected tadpole and adult frog intestines (**Figure 4A**). Healthy (mock-infected) tadpoles possessed significantly greater *csf3r* gene expression in their intestines than adult frogs (**Figure 4A**). Following FV3 challenge, tadpoles significantly increased their intestinal expression of both *csf1r* and *csf3r* genes while FV3-infected adult frogs increased their intestinal expression of *csf3r* but not *csf1r* (**Figure 4A**). Notably, FV3-infected tadpoles possessed significantly greater levels of both receptor gene transcripts in their intestines than seen in the intestines of FV3-infected adult frogs (**Figure 4A**).

Akin to their mammalian counterparts, the respective CSF1R and CSF3R ligands, CSF-1/IL-34 and CSF3 are chemotactic (27, 30). Accordingly, we examined the expression of *csf1*, *il34* and *csf3* genes in mock- and FV3-infected tadpole and adult intestines to account for the increased presence of myeloid cells in virally challenged tadpole but not adult intestinal tissues (**Figure 4B**). Compared to their mock-infected controls, FV3-infected tadpoles possessed significantly more transcripts of both *csf-1* and *il-34* in their intestines while adult frogs did not exhibit discernible changes in the expression of these genes (**Figure 4B**). The expression of the *csf3* gene in tadpole and adult intestines was not significantly altered by viral infections (**Figure 4B**). This corroborates our observations that the FV3-infected tadpoles possessed monocyte-like cells, but not granulocytes within their intestinal tissues (**Figure 3**).

To explore the possibility that the recruitment of the specific esterase-positive myeloid cells into infected tadpole intestines is mediated by other chemo-attractants besides CSF-1 and/or IL-34, we examined mock- and FV3-infected tadpole intestines for the expression of a panel of chemokine genes (**Supplementary Figure 2**). With the exception of *ccl20*, none of the examined chemokine genes were differentially expressed between mock- and FV3-infected tadpole intestines (**Supplementary Figure 2**). The *ccl20* chemokine gene showed variable and a non-significant expression increase in the FV3-infected tadpole intestines compared to mock controls (**Supplementary Figure 2**).

### FV3-Infected Tadpole Intestines Contain Cells That Are Chemoattracted by rIL-34 and rCSF-1

**Figure 5:**
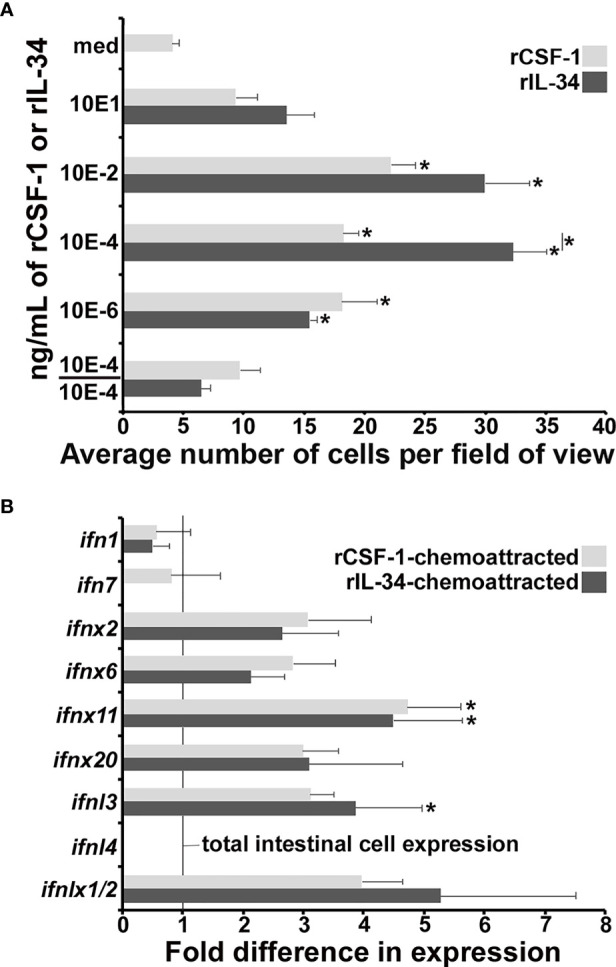
Recombinant CSF-1 and IL-34 elicit chemotaxis of *ifn*-expressing cells from FV3-infected tadpole intestines. Tadpoles (*N*=6) were infected by water bath with 10^6^ PFU of FV3 for 6 hrs, their intestines isolated and dispersed into cell suspensions. **(A)** These cells were used in chemotaxis assays, performed against medium alone (med) or 10 - 10^-6^ ng/ml of rCSF-1 or rIL-34 in bottom Boyden chamber wells (5 mm^2^; *N*=3 for medium, 10, 10^-2^, 10^-6^ ng/ml doses and *N*=9 for the 10^-4^ ng/ml doses of either cytokine) loaded into top wells, separated by chemotaxis filters. After incubation the bottom faces of the filters were stained with Giemsa stain and examined for numbers of migrating cells (40x objective). Chemokinesis experiments were performed using 10^-4^ ng/ml of rCSF-1 or rIL-34 (the most chemo-attractive concentration) in both bottom and the top chemotaxis chambers (*N*=3). The results are means ± SE of cells per field of view. Asterisks (*) denote statistical differences between medium alone and recombinant cytokine-induced migration (*) denote statistical differences between the treatment groups denoted by the line, *p*<0.05. **(B)** Cells chemoattracted by 10^-4^ ng/ml of rCSF-1 or rIL-34 (*N*=6) were examined for their type I and III IFN expression relative to *gapdh* endogenous control. The results are means ± SE of gene expression. Asterisks (*) denote statistical differences from the respective *ifn* expression in total intestines, denoted by line, *p*<0.05.

Because CSF-1 and IL-34 are chemotactic to macrophage-lineage cells (27, 30), we used recombinant forms of (r)CSF-1 and rIL-34 to see if the FV3-infected tadpole intestines contained cells that would migrate towards either cytokine. To this end, we performed chemotaxis assays on cell suspensions derived from FV3-infected tadpole intestines and using 10, 10^-2^, 10^-4^, 10^-6^ ng/mL of rCSF-1 or rIL-34 (**Figure 5A**). As anticipated based on our *csf1*/*il34* gene expression (**Figure 4B**), rCSF-1 and rIL-34 both chemoattracted intestine-derived cells in a concentration-dependent manner (**Figure 5A**). Notably, the 10^-4^ ng/mL of rIL-34 recruiting significantly more chemotaxis that the corresponding dose of rCSF-1 (**Figure 5A**).

To confirm that these migration responses were rCSF-1/rIL-34 gradient-dependent and not due to random migration (chemokinesis), we loaded optimal doses (10^-4^ ng/mL) of rCSF-1 or rIL-34 into upper and lower chemotaxis chambers (10^-4^/10^-4^), thereby abolishing the r-cytokine gradients, and again examined intestinal cell migration. Under these conditions, the cell migration was reduced to levels that were not significantly different from the media (med) controls (**Figure 5A**), indicating that the observed migration elicited by both rCSF-1 and rIL-34 were gradient-dependent chemotaxis.

### Cells Recruited by rCSF-1/rIL-34 From Infected Tadpole Intestines Possess Robust IFN Gene Expression

Because CSF-1 and IL-34 both ligate the CSF-1R (31), either rCSF-1 or rIL-34 will engage the CSF-1R on IL-34-macrophages, which we postulated to be responding to the tadpole intestinal FV3 infections. As such, we examined the infected tadpole intestinal cells chemotaxed to rCSF-1 and rIL-34 (10^-4^ ng/mL) for their expression of a panel of *ifn* genes (**Figure 5B**). Compared to total intestinal cells (vertical line in **Figure 5B**), these rCSF-1- and rIL-34-recruited cells exhibited greater expression of several of the examined *ifn* genes, with rCSF-1-chemotaxed cells expressing significantly greater levels of *ifnx11* and rIL-34-chemotaxed cells bearing significantly greater *ifnx11* and *ifnl3* mRNA levels (**Figure 5B**). Notably, neither rCSF-1- or rIL-34-recruited cells expressed *ifnl4* (**Figure 5B**), the expression of which was significantly upregulated in the FV3-infected tadpole intestines (**Figure 2C**).

### Specific Esterase-Positive Cells Populate the Frog Intestines During Metamorphosis

We reasoned that since adult frogs already possess specific esterase-positive cells in their intestines and appear to effectively contain FV3 replication (**Figure 1B**) with relatively modest IFN responses (**Figure 2**); the adult frog intestines must possess other antiviral effector mechanisms. To examine this notion, we compared the intestines of tadpoles, metamorphic, and adult frogs for their respective expression of a panel of antiviral genes including *pkr*, *mx1*, *apobec2*, *rad21*, *trim28* and *ifnar2.1* (**Supplementary Figure 3**). Of these examined genes, the restriction factor *apobec2* gene exhibited increased (not significant) expression in metamorphic frogs and significantly greater expression in adult frog intestines compared to those of tadpoles (**Supplementary Figure 3**). All of the other examined genes showed a non-significant trend towards greater expression in adults compared to tadpoles (**Supplementary Figure 3**).

**Figure 6:**
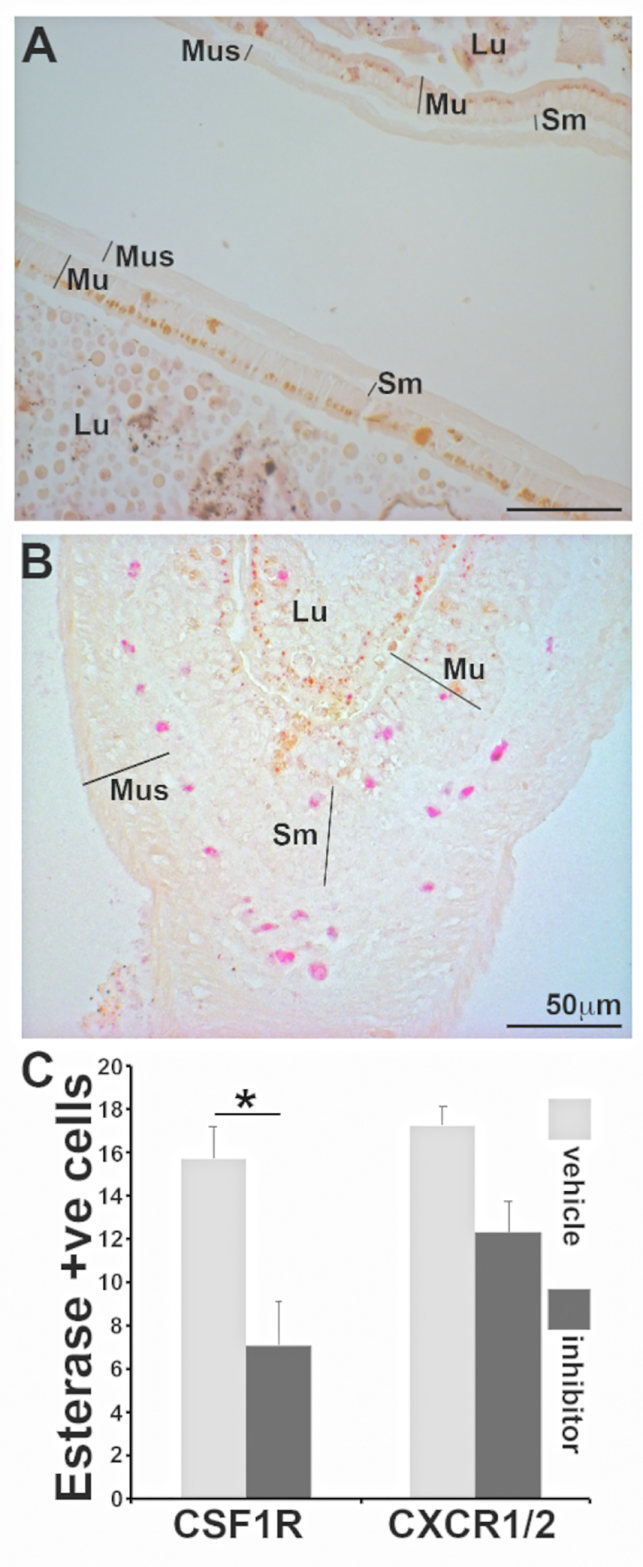
Frog intestines are populated my esterase positive myeloid cells during metamorphosis in a CSF1R-dependent manner. Representative images of intestines from **(A)** vehicle control (NaOH) and **(B)** T3 (10 nM final concentration)-treated tadpoles were examined by NASDCl-specific esterase (Leder) stain. Lu: lumen; Mu: mucosal; Sm: sub-mucosal r; Mus: muscularis layers. **(C)** Means ± SE (*N*=6 per treatment group) of specific-esterase positive cells (per field of view) in T3 (10 nM final concentration)-treated tadpoles administered with a vehicle control (DMSO in saline), a CSF1R inhibitor (GW-2580; 100 mg/kg body weight) or a CXCR1/2 inhibitor (reparixin; 50 mg/kg body weight). Asterisks above a line (*) denote statistical differences between the treatment groups denoted by the line, *p*<0.05.

*X. laevis* metamorphosis is initiated in response to thyroid hormone and the onset of metamorphosis may be artificially induced by exposing tadpoles to triiodothyronine **[**T3 (21)]. To confirm whether the specific esterase-positive cells populate the frog intestines upon onset of metamorphosis, we exposed tadpoles to T3 or vehicle control (NaOH), and examined their intestines for specific esterase-positive myeloid cells, as above. As expected, treatment of tadpoles with T3 resulted in the characteristic thickening of their intestines seen during natural metamorphosis as well as recruitment of specific esterase-positive cells into the mucosa, submucosal, and muscularis layers of their intestines (**Figures 6A, B**).

We recently showed that specific esterase positive granulocytes are homed into *X. laevis* skin *via* the CXCL8a/b-CXCR1/2 chemokine axis (18). To examine whether the homing of specific esterase-positive cells into metamorphosing frog intestines was due to CXCL8a/b or the result of CSF-1R activation (by CSF-1 and/or IL-34), we again exposed tadpoles to T3, treating subsets of animals with either a vehicle control, a pharmacological inhibitor of CSF-1R (GW-2580) or an inhibitor of the CXCL8a/b receptors [CXCR1/2: reparixin (18)]. Only CSF-1R inhibition resulted in a significant reduction of specific esterase-positive cells (**Figure 6C**). This suggests that homing/population of *X. laevis* intestines with these immune cells is mediated *via* the CSF-1 and/or IL-34 activation of CSF-1R during metamorphosis.

## Discussion

In order to slow and counteract the global amphibian declines caused by ranaviruses such as FV3, we must gain greater understanding of the mechanisms governing immunological susceptibility and resistance to these pathogens. The present manuscript represents a contribution towards this goal by elucidating the differences between intestinal anti-FV3 responses of the anuran *X. laevis* tadpoles and adult frogs. Indeed, it has been known for some time that FV3 targets amphibian intestines as part of its infection strategy (32). Here we show that compared to adult frogs, *X. laevis* tadpoles, which are thought to be significantly more susceptible to this pathogen (9), undergo distinct responses to intestinal FV3 exposure, marked by recruitment of myeloid cells into the infected tissues and upregulation of antiviral *ifn* gene expression therein.

In line with our previous reports of other infected tadpole and adult frog tissues (5, 11), our present findings indicate that infected tadpoles harbor lower FV3 loads in their intestines than adult frogs. Moreover, we observed tadpoles rely on the recruitment of specific esterase-positive mononuclear phagocytes into their intestines to control FV3 infections, though adult frogs possess resident esterase-positive cells and do not appear to be dependent on incoming myeloid-lineage immune cells for their control of intestinal FV3. Notably, while adult frogs possessed greater FV3 DNA loads in their intestines than tadpoles, the adult frogs were also host to substantially lower FV3 gene expression than infected tadpoles. Tadpole intestines comprise of a single layer of epithelial cells, with limited connective and muscle tissues (33). During metamorphosis, the tadpole intestinal epithelial cells undergo extensive apoptosis, concurrent with the proliferation of adult epithelia, thickening of muscle and mesenchyme, and ultrastructure formation reminiscent of that seen in higher vertebrates (33–35). The difference in FV3 infection and replication within the tadpole and adult intestines undoubtedly reflects these cellular and physiological differences. Presumably, the adult frog intestinal cells are less permissive to FV3 replication despite greater viral entry and/or accumulation therein. Possibly, adult frogs possess a greater proportion of intestinal cells that are susceptible to FV3 entry than tadpoles, while the immune cells residing in the adult, but not tadpole intestines curb viral replication at the onset of infection. This may in turn relieve adult frogs from needing to recruit additional immune cells into their infected intestines or mounting robust IFN responses, as seen in tadpole intestines. Moreover, adult frogs expressed greater levels of the gene encoding the APOBEC2 restriction factor in their intestines, which may also account for their greater resistance to FV3 replication. That FV3 persists in the adult frog intestines without infiltrating intestinal cells is a possible, although unlikely explanation that is further negated by data from other laboratories (32) and our own unpublished observations indicating FV3 may remain in the adult frog intestines for over a week.

Tadpole *ifn* gene expression responses to intestinal FV3 infections were considerably more prominent than those detected in the adult frogs. Since anuran amphibians like *X. laevis* have highly expanded repertoires of intronless and intron-containing type I and type III IFN genes with likewise potentially expanded functional diversity (15, 16), it is perhaps intuitive that tadpoles and adult frogs make distinct use of these antiviral cytokine repertoires (10–12). Our observation that adult frogs are much better at controlling FV3 gene expression than tadpoles may explain why the adult frogs mount less robust *ifn* gene responses to FV3, as greater antiviral control and thus lower viral products in adult intestinal cells would likely result in less immune receptor activation and thus less *ifn* gene expression by virally-infected or bystander cells.

Notably, our preliminary analyses of FV3-infected tadpole and adult frog intestinal *ifn* gene expression indicated that majority of the tadpole *ifn* responses initiated at 6 hours of infection. Thus, in the present work, we chose to focus on this infection timepoint to discern the early differences between tadpole and adult frog intestinal anti-FV3 responses. In addition to our findings, there are presumably further differences in the immune strategies employed by tadpoles and adult frogs in dealing with ranavirus infections within distinct tissues and at distinct infection times. Future studies that extend upon our findings and compare tadpoles and adult frogs at later FV3 infection times will undoubtedly provide further insight into the developmental stage-dependent differences in antiviral immunity. Moreover, further characterization of the myeloid cells recruited into FV3-infected tadpole intestines and residing within healthy adult frog intestines will grant a better understanding of the differences in ranavirus susceptibility seen between anuran tadpoles and adult frogs.

Interestingly, we did not observe significant differences between the mock-infected tadpole and adult frog *ifn* gene expression in their respective intestinal tissues, suggesting that the adult frog intestine-resident specific esterase-positive cells are not rendering these tissues more resistant to FV3 replication through IFN production. Possibly, these and other resident adult frog immune cells are producing other antiviral mediators that prime intestinal cells against viral replication. Alternatively or in parallel, the adult frog intestinal cells may be inherently more resistant to FV3 replication, perhaps due to greater baseline expression of interferon-stimulated genes, other restriction factors such as APOBEC2, and/or their ability to recruit other effector cells.

The paucity of knowledge regarding tadpole and adult frog immune compositions within ranavirus-targeted tissues makes it difficult to discern whether *ifn*-expression differences described here are due to tissue cell composition and/or developmental stage-dependent constraints for mounting specific IFN responses. It is notable that the FV3-infected tadpole intestinal *ifn* gene responses coincided with increased *il34* and *csf1* gene expression and the appearance of specific esterase-positive cells bearing IL-34-macrophage-like morphology. We previously demonstrated that *X. laevis* IL-34-macrophages, but not CSF-1-macrophages, possess robust specific esterase activity along with monocyte-like morphology and are important IFN producers (19, 28, 30). Notably, CSF-1 renders macrophages significantly more susceptible to FV3 (28), so the greater FV3 gene expression detected in the tadpole compared to the adult frog intestines may be stemming at least in part from an increased presence of CSF-1-macrophages. Alternatively, it is possible that both tadpole and adult frog intestinal cells are equally non-permissive to FV3 replication, and the decreased *in vivo* FV3-loads in tadpole intestines are due to greater presence of IL-34-macrophages, while the greater FV3 gene expression reflects the recruitment, expansion and/or polarization of CSF-1 macrophages at this site.

Our results indicate that the specific esterase-positive phagocytes that infiltrate FV3-infected tadpole intestines respond to rCSF-1 and rIL-34 and express some, but not all of the *ifn* genes that are upregulated within infected tadpole intestines. As discussed above, our past work indicates *X. laevis* IL-34-macrophages, but not CSF-1-macrophages, are robust IFN producers (28), while our present work indicates FV3 elicits increased expression of both *csf1* and *il34* genes in the tadpole intestines. Thus, we anticipate both macrophage populations are present within the FV3-infected tadpole intestines, and because both of these subsets express CSF-1R (19), both would respond to either rCSF-1 or rIL-34. As such, the cells chemoattracted out of the infected tadpole intestines by either rCSF-1 or rIL-34 likely contain both CSF-1- and IL-34-macrophages. We anticipate only the IL-34-macrophages account for the increased *ifn* gene expression in the infected intestines, so a mixed chemoattracted CSF-1-/IL-34-macrophage population would differ in their *ifn* gene expression from total intestinal *ifn* expression. Moreover, we speculate some of the examined *ifn* genes may be expressed by other, presently unknown incoming immune subsets and/or elicited by factors produced by IL-34-macrophages and/or these other leukocytes in the tadpole intestinal cells.

While tadpoles and adult frogs clearly possess drastically distinct physiologies and susceptibilities to pathogens like FV3, the immune and non-immune cell compositions of the respective organs targeted by this virus remain poorly understood. Concurrently, the immune responses elicited in tadpoles and adult frogs by FV3 require further investigation. Pathogens like FV3 have co-evolved with their amphibian hosts and studying the differences in the tadpole and adult frog immune responses to such pathogens will grant novel perspectives of how the evolution of host-pathogen interactions may be shaped by developmental stage-dependent differences in immune capabilities and strategies.

## Data Availability Statement

The raw data supporting the conclusions of this article will be made available by the authors, without undue reservation.

## Ethics Statement

The animal study was reviewed and approved by IACUC Approval number 15-024.

## Author Contributions

KH, JS, and LG designed and planned the studies. KH, JS, MH, TM, EW, AY, NK, and LG performed the experiments. KH, JS, and LG analyzed the data, wrote the manuscript, and prepared the figures. All authors contributed to the article and approved the submitted version.

## Funding

This work was supported by a National Science Foundation CAREER Award (IOS: 1749427) to LG.

## Conflict of Interest

The authors declare that the research was conducted in the absence of any commercial or financial relationships that could be construed as a potential conflict of interest.

## Publisher’s Note

All claims expressed in this article are solely those of the authors and do not necessarily represent those of their affiliated organizations, or those of the publisher, the editors and the reviewers. Any product that may be evaluated in this article, or claim that may be made by its manufacturer, is not guaranteed or endorsed by the publisher.

## Supplementary Material

Supplementary Figure 1 Non-specific esterase stain of mock- and FV3-infected tadpole intestines. Lu: lumen; Mu: mucosal; Sm: sub-mucosal r; Mus: muscularis layers. Images are representative of results derived with 5 individual animals per treatment group.

Supplementary Figure 2 Analyses of (A) CC- and (B) CXC-motif chemokine genes in mock- and FV3-infected tadpole intestines. (C) Comparison of *ccl20* expression in mock- and FV3-challenged tadpole and adult intestines. Results are means ± SE of gene expression relative to *gapdh* endogenous control (*N*=6). Asterisks above lines (∗) denote statistical differences between the treatment groups denoted by the lines, *p*<0.05.

Supplementary Figure 3 Comparison of antiviral gene expression in the intestines of tadpoles (stage NF 54), metamorphic (stage NF 62) and adult frogs. Results are means ± SE of gene expression relative to *gapdh* endogenous control (*N*=6). Asterisk (∗) denotes statistical difference from tadpole expression, *p*<0.05.
