# Supplementary figures and images for: Amphibian (Xenopus laevis) Tadpoles and Adult Frogs Differ in Their Antiviral Responses to Intestinal Frog Virus 3 Infections
Source: Front Immunol. 2021 Aug 20;12:737403. doi: 10.3389/fimmu.2021.737403 (PMC8418544; doi:10.3389/fimmu.2021.737403)

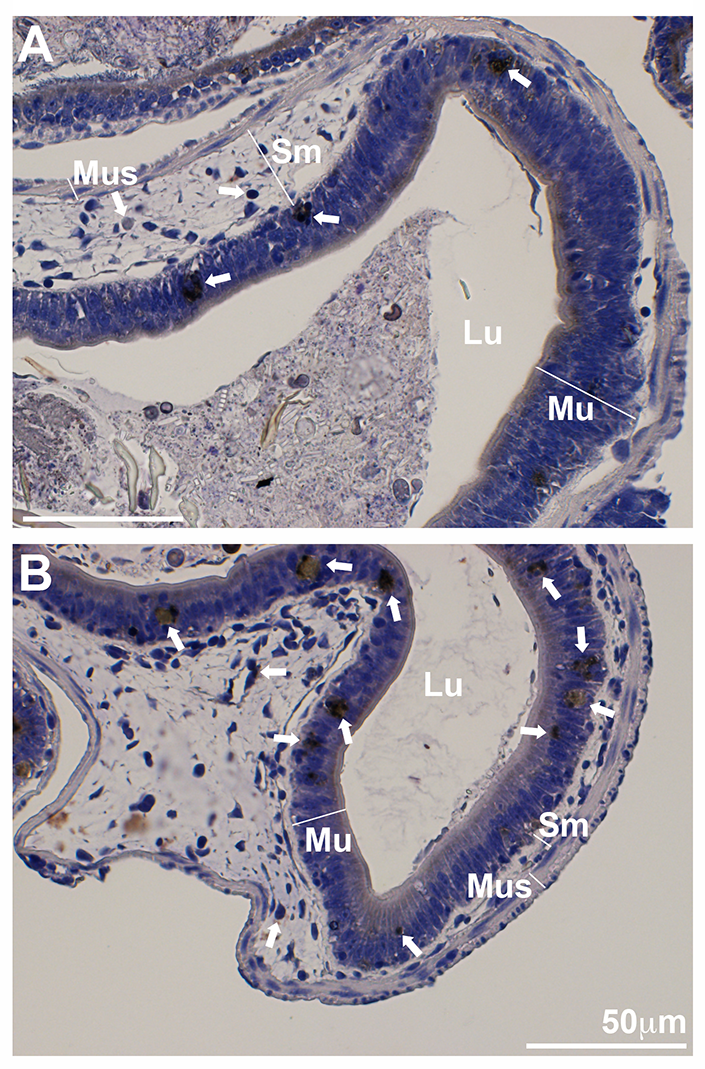

Supplement: Supplementary Figure 1 — Non-specific esterase stain of mock- and FV3-infected tadpole intestines. Lu: lumen; Mu: mucosal; Sm: sub-mucosal r; Mus: muscularis layers. Images are representative of results derived with 5 individual animals per treatment group. [file Image_1.tif]

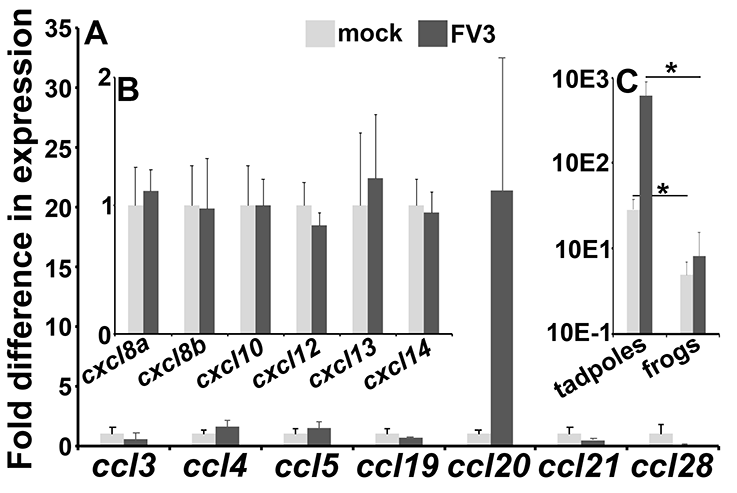

Supplement: Supplementary Figure 2 — Analyses of (A) CC- and (B) CXC-motif chemokine genes in mock- and FV3-infected tadpole intestines. (C) Comparison of ccl20 expression in mock- and FV3-challenged tadpole and adult intestines. Results are means ± SE of gene expression relative to gapdh endogenous control (N=6). Asterisks above lines (∗) denote statistical differences between the treatment groups denoted by the lines, p<0.05. [file Image_2.tif]

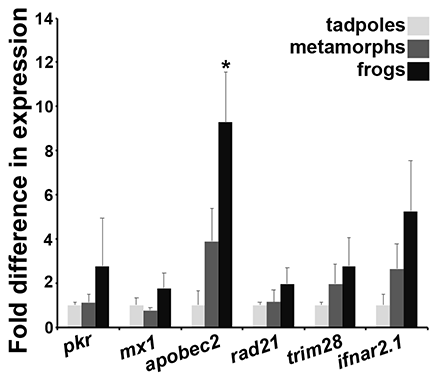

Supplement: Supplementary Figure 3 — Comparison of antiviral gene expression in the intestines of tadpoles (stage NF 54), metamorphic (stage NF 62) and adult frogs. Results are means ± SE of gene expression relative to gapdh endogenous control (N=6). Asterisk (∗) denotes statistical difference from tadpole expression, p<0.05. [file Image_3.tif]
