# Supplementary material for: Amphibian (Xenopus laevis) Tadpoles and Adult Frogs Differ in Their Antiviral Responses to Intestinal Frog Virus 3 Infections
Source: Front Immunol. 2021 Aug 20;12:737403. doi: 10.3389/fimmu.2021.737403 (PMC8418544; doi:10.3389/fimmu.2021.737403)
Supplement: Supplementary file 4 [file Table_1.docx]

**Supplementary Table 1.** List of primer sequences

| **Primer** | **Sequence 5’🡪3’** | **Primer** | **Sequence 5’🡪3’** |
| --- | --- | --- | --- |
| *apobec2* F  *apobec2* R | CCCTGCTTCTTCTTTCATGTTTC  TATTTGAGCCTCAGGTCTTTCC | *FV3 82R* F  *FV3 82R* R | TGTGTCTGGAGAACCCTACA  TGGTGATCTTGACTTGAAACTC |
| *ccl3* F  *ccl3* R | AGTGTGGACCAGGAGGTAA  CTGCTGGTGATATAATAGTCCTTGA | *FV3 93L* F  *FV3 93L* R | GCCAAGACCGAGGACAC  GGACAAACCAACAGTAGAAACG |
| *ccl4* F  *ccl4* R | TCCAACCCTGGCGTTATATTC  GCATTCTTGTCCAGCTTTGTC | *FV3 95R* F  *FV3 95R* R | CATGTCAACGTCAAAGTCCAAG  TGTGCACCCTCTGGTTAAAG |
| *ccl5* F  *ccl5* R | GTTCTTGTCACCCGGAAGAA  TGTGTTGCTTCAGGCATCT | *gapdh* F  *gapdh* R | ATGTGTCCGTTGTGGATCTG  GATTCCTTTCATTGGTCCCTCT |
| *ccl19* F  *ccl19* R | ACCATCAACAAGCCGATTCC  GAGCTGTATGTTTCTCCGAGTG | *ifn1* F  *ifn1* R | ACCTGTCGAGGTTGCATATTAG  GCAATGAGGGTGCAGAATTAG |
| *ccl20* F  *ccl20* R | CTCCTGGCTGCTTTGATGT  GGATGAAGAATACGGTCTGTGTAT | *ifn7* F  *ifn7* R | TCTGTAGGAAGTCTCCGAAGTA  CACATTCAGTTGGAACCCTTTC |
| *ccl21* F  *ccl21* R | ATCCAGTGGCTGCTCAATC  CTGGCAATCATAGTCTGTACCC | *ifnar2.1* F  *ifnar2.1* R | CTGTTCTGTTAAAGTCTCACTA  CTTGGGTGGACGAAGTGGT |
| *ccl28* F  *ccl28* R | GCTCTCTGTGTCAATCCCAATA  CCGGTGTTTCTTTCTTCCCT | *ifnl3* F  *ifnl3* R | GTCCTTTCAGCGATGGGATAA  ACGGCTTATGGCGAAACA |
| *csf1* F  *csf1* R | CTCTGTCCAAGCTGGATGATT  GCCTTATTCATCCCATGTGTTTC | *ifnl4* F  *ifnl4* R | TCTGTAGGAAGTCTCCGAAGT  CACATTCAGTTGGAACCTTT |
| *csf3* F  *csf3* R | AGGCATAACAGCCCAGAAC  GTCGGAAAGGATGAGGAACAT | *ifnlx1/2* F  *ifnlx1/2* R | TGACCACCTGGTTACCTACA  CCAGGAATCTCATGTCCTCAATC |
| *csf1r* F  *csf1r* R | GAGCAAGGGCACTGATAGTT  AAAGGTCTACGGGCAAGATG | *ifnx2* F  *ifnx2* R | TCCATGCTTGTCTGCACAT  ACCCTAGCACAGATGGGA |
| *csf3r* F  *csf3r* R | TGGATGAAGGACTACAGCTAATG  GCCTGTCATCTGTGAGGTTTA | *ifnx6* F  *ifnx6* R | CAGTCCATCTCCTCACACTAAC  CGCTTGATCCTGTGTCTTGTA |
| *cxcl8a* F  *cxcl8a* R | CATAAAGACAGAAAGCAAGCCT  GGCTCCAAGCAGATATCG | *ifnx11* F  *ifnx11* R | CATGCCAACAACTGGTTTCTC  AATGCTGAGCCTCTGAAGATT |
| *cxcl8b* F  *cxcl8b* R | ACGACCCACTCTGCTTTC  TTTCTACCCAGCGCTGAG | *ifnx20* F  *ifnx20* R | CATGGTGGTGCATACAGTCTAC  CCTCCAGAAGGGAACGTAGATA |
| *cxcl10* F  *cxcl10* R | GGCTGTGATGAAGTTGAAGTTG  GTAGGGCAGTCATGAGTTTGT | *il34* F  *il34 R* | CCAAGGAATCACCGTAGAAGAG  GGTGTCTGTCTGGCAATACTT |
| *cxcl12* F  *cxcl12* R | ATAAAAAACACATTCGTTCCT  ACAGCGGTTAACCACTTGG | *mx1 F*  *mx1 R* | GAGCAGAGTCAGATGTTGTCAG  CAGCGAAAGGTTCTCCTGTATC |
| *cxcl13* F  *cxcl13* R | AAGCTGACACGGGTTGAA  CAGCTTGTGGATTGACACATAC | *pkr F*  *pkr R* | GATCTCAGATGTCCGAGTTTGT  TCTGGTTTCTGGCTCTCTAAAC |
| *cxcl14* F  *cxcl14* R | GGACTTGTACTGCCGTTCTT  GCGTACCTTCCCATGTAATGA | *rad21* F  *rad21* R | CATGACTTTGACCAGCCTCTAC  GTTGCTAACCTCTTCCCTCATT |
| *vDNAPol* F  *vDNAPol* R | CAAGAACGTGTGCTACTCCA  AGCCTCTCGTACTCTACCTTC | *trim28* F  *trim28* R | CACATGTGGCCAGTGTAGAA  GAGGTGAGATCCTGGCATAAC |
